# 蛋中全氟/多氟化合物的分析方法及其作为污染指示物的应用

**DOI:** 10.3724/SP.J.1123.2020.09023

**Published:** 2021-02-08

**Authors:** Tong YE, Yu CHEN, Jie FU, Aiqian ZHANG, Jianjie FU

**Affiliations:** 1.江汉大学环境与健康研究院, 湖北 武汉 430010; 1. Institute of Environment and Health, Jianghan University, Wuhan 430010, China; 2.中国科学院生态环境研究中心, 环境化学与生态毒理学国家重点实验室, 北京 100085; 2. State Key Laboratory of Environmental Chemistry and Ecotoxicology, Research Center for Eco-Environmental Sciences, Chinese Academy of Sciences, Beijing 100085, China; 3.国科大杭州高等研究院环境学院, 浙江 杭州 310000; 3. School of Environment, Hangzhou Institute for Advanced Study, UCAS, Hangzhou 310000, China

**Keywords:** 蛋, 生物指示物, 样品前处理, 全氟/多氟化合物, eggs, bioindicator, sample pretreatment, perfluoroalkyl and polyfluoroalkylated substances (PFASs)

## Abstract

全氟和多氟化合物(perfluoroalkyl and poly-fluoroalkylated substances, PFASs)在环境中有极强的持久性和生物富集能力,在全球生态系统及人体中已被普遍检出,其潜在的毒性引起了人们高度关注。蛋是卵生动物生命的起源,同时其营养丰富,是人类主要的蛋白质来源之一,因此,蛋中的污染物水平一方面有可能影响卵生动物种族繁衍,另一方面也关系到人类的健康风险。近年来禽蛋类样品作为非损伤性生物基质已广泛应用于生物体和生态系统污染情况的评估,同时利用蛋类样品中污染物水平评估相应的遗传发育毒性风险和摄入健康风险的研究也逐渐增多。该文对现有文献中蛋中PFASs的样品前处理和仪器检测方法进行了归纳总结,并且对蛋作为PFASs污染指示物的适用性和先进性进行了讨论。

## Abstract (en)

Perfluoroalkyl and polyfluoroalkylated substances (PFASs) are environmentally persistent and biomagnified along food chains. They have been widely detected globally, even in the human body, and their potential toxicity has attracted great attention. Eggs are the origin of new life of ovipara and are rich in nutrients, thus they serve as one of the main protein sources for humans. Therefore, the level of pollutants in eggs can affect the reproduction of ovipara, and it is also related to human health by food intake. In recent years, poultry egg samples have been widely used in the assessment of biological and ecological pollution as a non-invasive biota matrix. At the same time, recent studies have used eggs to evaluate the developmental toxicity and associated health risks based on the pollutant levels in egg samples. In this study, the methods of sample pretreatment and instrumental detection of PFASs for egg samples are summarized. In addition, the application of eggs as a pollutants bioindicator of PFASs contamination has been discussed.

全氟和多氟烷基化合物(perfluoroalkyl and poly-fluoroalkylated substances, PFASs)是一类烷基中的氢原子被多个或全部氟原子取代的有机化合物。PFASs按照末端基团的不同可以分为不同种类:包括全氟烷基羧酸类(perfluorinated carboxylate acids, PFCAs)、全氟烷基磺酸类(perfluoroalkyl sulfonic acids, PFSAs)、磺酰胺类(fluorooctane sulfonamides, FOSAs)、氟化调聚醇(fluorotelomer alcohols, FTOHs)、全氟膦酸(perfluorinated phosphonic acids, PFPAs),及其酯(PAPs)等(见图1)。PFASs中碳氟键键能极高(约484 kJ/mol),使得它具有很强的稳定性,并且碳氟链具有疏水疏脂及高表面活性等特点^[1]^。PFASs被广泛应用于工业生产及生活用品等多个领域,如化工、食品包装、泡沫灭火剂等^[2,3]^。早在20世纪40年代,美国3M公司采用电化学氟化法先后生产了全氟辛酸(perfluorooctanoic acid, PFOA)^[4]^和全氟辛烷磺酸(perfluorooctanesulfonate, PFOS)的原料全氟辛烷磺酰氟(perfluorooctane sulfonyl fluoride, PFOSF)等典型全氟化合物,长期以来是全球最大的PFOS相关产品的制造商,大约生产了世界上85%的PFOS^[5]^。据Paul等^[6]^估计,全球PFOSF在1970年到2002年间的历史总产量约为122500吨(包括相关副产物和废物),其中通过制造、使用、处置和前体化合物及副产物的排放等方式向空气和水中释放的PFOSF含量为45250吨。PFASs在环境中表现出来的持久性和生物富集能力及一定的毒性^[7]^对生态系统及人类健康造成严重威胁,受到了科学家和公众越来越多的重视。Giesy和Kannan在2001年首次发现PFOS在全球范围内的鱼类、鸟类以及哺乳动物中普遍存在,即使南极和北极等地区生物也不例外^[8]^。后续的研究表明,PFASs在大气、水体等环境介质^[9]^以及人体中^[10]^也普遍存在,是一类全球性的污染物。PFOS和PFOA及其相关前驱体已经被列入斯德哥尔摩公约限制生产使用,全氟己烷磺酸(perfluorohexane sulfonic acid, PFHxS)也已进入公约审查名单^[11]^。

**图1:**
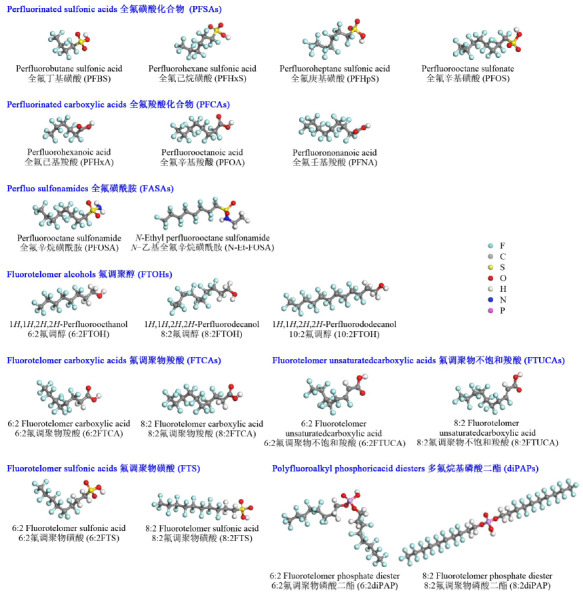
部分PFASs的分类、名称及化学结构

PFASs在不同环境介质中的分布并不均一,甚至在同一生态系统的不同环境介质中浓度差距可达几个数量级^[12,13,14]^。不同研究中采用的环境样品并不一致,导致了不同研究区域之间PFASs污染水平的相互比较存在困难,不利于评估其整体风险。选择一种跨区域普遍存在、容易采集的环境样品进行检测,可使不同区域PFASs污染状况的横向比较和风险评估成为可能。水的化学成分单一,采样较为简单,是一种比较理想的研究介质,但环境水体中一般污染物浓度很低,需要大量采集,运输不便,并且其比较容易受到点源污染的干扰。鉴于一些污染物在生态系统中具有显著的生物放大能力^[15,16]^,选择生物样品作为研究基质可以更好地展示区域污染状况,同时通过生物中污染物的浓度来评价其生态风险也更为直接和有效。PFASs与转运蛋白具有较强的亲和力,导致其在转运蛋白含量较高的血液和肝脏中浓度偏高,因此血液和肝脏样品是研究生物PFASs负荷的常见基质^[17,18,19]^。但肌肉组织或血液的采样对于动物本身具有侵害性,尤其在针对濒临灭绝的野生动物(国家保护动物)研究中,样品难以获取。因此近年来大量的研究开发了非侵害性采样技术,鸟类的羽毛、蛋(卵)都可作为非侵害性样品用于指示环境中的新型污染指示物^[20,21]^。羽毛样品便于采集和运输,但是一般认为羽毛中的污染物同时存在外源和内源,不能很好地反映生物体内污染物实际负荷^[20]^。与之相比,蛋(卵)中的污染物则全是来源于生物体内部,且不同鸟类在蛋之间成分较为一致,同时在运输过程中不易被污染^[22]^。蛋(卵)在受精后可直接孵化为子代生物,因此蛋(卵)中污染物水平与污染物代际传递相关联,可直接影响胚胎的发育^[23,24]^。基于以上特点,近年来对蛋(卵)中污染物水平的研究日益增多^[25,26]^。

与其他介质相比,蛋中脂质含量丰富,因此预处理时经常会出现除脂不彻底,在检测过程出现较强的基质干扰,有可能出现PFASs检出限较高,甚至造成色谱柱或质谱故障等情况,影响其作为PFASs污染指示物的应用。本文对现有文献中蛋中PFASs的前处理和仪器检测方法进行了总结,可为后续研究中对复杂基质中PFASs的提取和分析提供参考。同时,目前已有不少研究中报道了鸟类蛋中PFASs的水平,本文对利用鸟类蛋作为环境污染指示物的应用也进行了探讨。

## 1 蛋中PFASs的分析方法

通过检索Web of Science数据库,2000至2020年之间共有99篇文献研究了蛋中PFASs的赋存,我们对这些研究中的目标PFASs、前处理方法及分析检测方法进行了总结:目前蛋中所关注的主要PFASs为PFSAs、PFCAs及其前驱体;对于蛋中PFASs的萃取方法主要有离子对萃取(ion pair extraction, IPE)、碱消解(alkaline digestion method, ADM)和甲醇/乙腈直接提取等方法,净化方法主要有固相萃取、分散固相萃取等方法。PFASs的检测手段则主要为液相色谱-串联质谱法(LC-MS/MS)和气相色谱-串联质谱法(GC-MS/MS)。

### 1.1 样品前处理方法

样品前处理包括萃取、净化和浓缩等过程,可以有效地选择性富集目标化合物,降低基质效应,特别是对于蛋类这种基质复杂、PFASs浓度较低的生物样品,更需要选择合理的萃取方法来提高萃取效率和分析灵敏度。

1.1.1 离子对萃取

离子对萃取中被萃物质为疏水性离子缔合物,是被萃取物质与离子对试剂缔合后通过有机溶液进行萃取的一种方法。当前,该方法通常以四丁基硫酸氢铵(tetrabutylammonium hydrogen sulfate, TBA)作为离子对试剂,以甲基叔丁基醚(methyl tert-butyl ether, MTBE)作为萃取剂。Yoo等^[27]^为了评估PFOS和其他PFASs对韩国始华湖鸟类的生态风险,利用离子对萃取结合HPLC-MS/MS对鸟蛋蛋黄中的9种PFASs进行分析,平均回收率达到83%。Custer等^[28]^利用同样的方法对大蓝鹭(*Ardea herodias*)蛋中的11种PFASs进行萃取,样品加标回收率在68%±21%~120%±14%之间。该萃取方法操作简便、耗时较短,但经前处理后样品中仍有较明显的色素,因此基质效应较为明显,需要进一步优化调整。Su等^[29]^在用离子对萃取技术对鸡蛋中的PFASs进行萃取后,为了去除基质中的杂质和色素,先使用ENVI-carb固相萃取柱,后使用Oasis-WAX柱富集全氟烷基酸(perfluorinated alkyl acids, PFAAs),该方法中目标PFAAs的基质加标回收率为68%±1%~134%±7%。本研究组前期也通过该方法对鸡蛋中23种PFASs进行了分析测定,其中18种PFASs的回收率在53.17%~158.16%, 4种化合物回收率在30%左右,另有1种的回收率超出正常水平,达到了300%(未发表数据),这一方面可能是由于净化过程去除萃取过程中引入的脂质和基质干扰物不完全,另一方面可能是由于PFASs间物化差距大,该方法不适合部分PFASs的前处理。

1.1.2 碱消解

PFASs具有亲蛋白质的特性,富含蛋白质的基质样品在萃取过程中有可能产生蛋白质与溶剂竞争导致萃取不完全的情况。通过碱消解能彻底破坏蛋白质结构,完全释放与其结合的PFASs,因此,该方法经常用于生物样品中PFASs的萃取,常用萃取液为氢氧化钾(KOH)和氢氧化钠(NaOH)的甲醇或水溶液。So等^[30]^对比了不同浓度的KOH甲醇溶液和KOH水溶液的消解效果,发现碱性甲醇溶液(0.01 mol/L)对所有PFASs的回收率最好且回收率均大于70%;而碱性水溶液(0.3、0.5、1和2 mol/L)对于全氟十二烷基酸(perfluorododecanoic acid, PFDoDA)和全氟十一烷基酸(perfluoroundecanoic acid, PFUnDA)的回收率仅在2.1%~24.6%。虽然碱性甲醇溶液对于PFASs的萃取效果较好,但也有不一致的研究。Tahziz等^[31]^对马来西亚禽蛋蛋黄中的PFOS和PFOA的浓度进行测定,在前处理时,尝试了几种清洗和提取程序的组合:首先采用碱消解方法,在每个样品中加入2 mL 0.2 mol/L的NaOH甲醇溶液,随后用固相萃取法进一步净化富集,分别比较了Oasis WAX和Oasis HLB这两种固相萃取柱的净化富集能力,结果显示使用Oasis WAX及HLB净化添加基质(5 ng/g)的平均回收率均较低,分别为30%和32%;随后在此基础上,以煮熟的蛋黄样品为基质,经碱消解20min后进行超声萃取和净化等步骤,萃取效果依然较差,回收率仅在55%~60%,这可能是基质效应造成的,因为蛋黄具有较高的脂质百分比,如果没有高效的净化步骤,脂质和其他基质成分可能导致增强或抑制电喷雾电离,从而影响检测结果^[32]^。

1.1.3 甲醇/乙腈直接萃取

甲醇/乙腈作为萃取溶剂的时候通常配合分散固相萃取对生物样品中的PFASs进行分析,这在保证不受外源性污染的情况下,大大缩短了前处理时间。李静等^[33]^建立了以乙腈超声提取结合分散固相萃取净化、通过HPLC-MS/MS测定鸡蛋中16种PFASs的方法,实验表明加入50 mg石墨化碳黑净化时,16种PFASs的萃取效果最佳,回收率在72%~120%之间,其中长链羧酸类PFASs的回收率达到72%~100%。刘晓湾等^[34]^同样以乙腈为萃取液,利用分散固相萃取的方法对鸡蛋样品中17种PFASs进行萃取,在石墨化碳黑(graphitized carbon black, GCB)的基础上额外添加*N*-丙基乙二胺(*N*-(*n*-propyl)ethylenediamine, PSA)和C_18_对鸡蛋进行净化,回收率达到了81%~120%。Eriksson等^[35]^在乙腈萃取的基础上,通过添加50 mg石墨化碳黑和100 μL冰醋酸优化前处理方法,对鱼鹰(*Pandion haliaetus*)、红隼(*Falco tinnunculus*)、灰林鸮(*Strix aluco*)蛋中PFCAs、PFSAs、氟调聚物不饱和羧酸(fluorotelomer unsaturated carboxylic acids, FTUCAs)、氟调聚物羧酸(fluorotelomer carboxylic acids, FTCAs)、氟调聚物磺酸(fluorotelomer sulfonic acids, FTSAs)、FOSAs、氨基乙醇类(perfluorooctane sulfonamido-ethanol, FOSEs)以及多氟烷基磷酸二酯(polyfluoroalkyl phosphoric acid diesters, diPAPs)等20余种PFASs进行检测分析,除了全氟辛烷磺酰胺(perfluorooctane sulfonamide, PFOSA)的回收率为38%,其余PFASs的平均回收率在83%~129%。该前处理方法对蛋类样品中长碳链羧酸类PFASs的回收率较其他方法高,并且具有耗时少、成本低等优点,但值得注意的是,为了除去脂肪酸、脂肪、色素等杂质而加入的石墨化碳黑,因其对平面性分子具有很强的吸附性也可能导致在净化过程中同时吸附目标待测物从而影响检测结果。

1.1.4 其他萃取方式

除以上几种前处理方法外,近年来也出现了一些新的改进方法,用于蛋类中PFASs分析的前处理。Tahziz等^[31]^以简单的蛋白质沉淀技术对鸡蛋样品中PFOS和PFOA进行萃取,该方法在Malinsky、Jacoby和Reagen^[36]^所运用的技术上进行了一些修改:在蛋黄样品中加入乙腈溶剂导致蛋白质聚集,在离心后,沉淀成颗粒状,将这些蛋白质颗粒去除后对萃取液进行干燥,接着加入甲醇转溶过滤后用LC-MS/MS测样,该方法中PFOS和PFOA的平均回收率在84%~102%之间。Letcher等^[37]^用酸消解方法,以甲酸乙腈为萃取液,配合固相萃取柱对银鸥(*Larus argentatus*)蛋中的PFSAs、PFCAs及FASAs进行净化,经检测后发现PFASs的内标回收率在47%±12%和91%±10%之间。Qi等^[38]^用QuEChERS法对长江和珠江三角洲地区收集到的鸡蛋和鸭蛋中的PFASs进行萃取,样品经盐酸乙腈萃取后,加入氯化钠(NaCl)盐析分层,随后采用C_18_、GCB、PSA等吸附剂与基质中绝大部分干扰物(有机酸、脂肪酸、碳水化合物等)结合,通过离心方式去除、净化,PFASs的回收率在70%~112%之间。

表1对蛋中PFASs的不同萃取方法进行了总结。虽然蛋类中PFASs的前处理方法较多,且各有优缺点,但鉴于蛋中蛋白质和脂含量丰富,PFASs又具有亲蛋白质的特性,因此对基质前处理时采用碱消解方法更为合适。另外,为了有效富集目标待测物、降低仪器分析过程中产生的基质效应,在样品萃取后,选择固相萃取技术对样品进一步净化也尤为必要。

**表1:** 蛋类基质中PFASs的分析方法

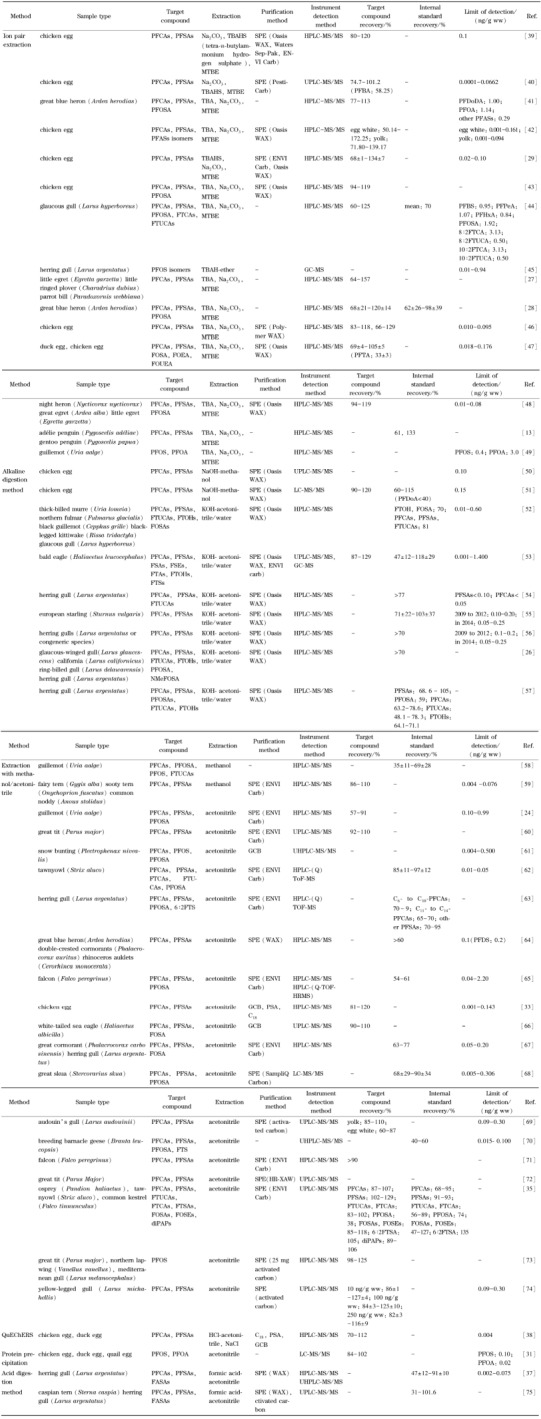

ww: wet weight; WAX: weak anion exchange; -: not available or not applicable.

### 1.2 仪器分析检测方法

1.2.1 液相色谱-串联质谱法

HPLC-MS/MS是最常用的PFASs检测方法,该方法能对环境及生物样品中的离子型PFASs进行准确定量检测^[76,77]^。三重四极杆串联质谱选择性和灵敏度高、线性范围宽、检出限较低,弥补了高效液相色谱-质谱(HPLC-MS)选择性差、基质干扰严重的缺点,在低浓度和基质较为复杂的情况下,优势尤其明显。高效液相色谱-串联飞行时间质谱法(HPLC/Q-TOFMS)尽管具有高分辨率和高质量准确度,减缓了基质干扰,但是Q-TOFMS灵敏度较低、线性范围窄,在实际环境样品中的应用较少。

电喷雾离子(ESI)源在低浓度样品和复杂基质样品检测过程中有着显著优势,是蛋类中PFASs检测用到的主要电离源。由于生物组织和大量内源性物质具有较高盐度和较多表面活性位点,会与待测物竞争液滴表面有限的电荷和空间,从而产生离子抑制,因此有时也采用大气压电离(APPI)源电离待测物^[78]^,尤其是对于中性全氟化合物,大多采用APPI源。Braune等^[52]^使用LC-ESI-MS/MS检测加拿大极地地区鸟蛋样品中的离子型全氟化合物,使用LC-APPI-MS/MS检测样品中的氟调聚醇和全氟磺酰胺类物质,APPI源能有效电离非极性或低电荷亲和力物质。Chu等^[79]^使用LC-APPI-MS/MS检测北极熊肝脏和海鸟蛋等生物样中的FTOHs和FOSAs,这两类物质的线性响应区间分别达到0~1000 ng/mL和0~250 ng/mL,首次证明了与ESI相比,APPI对FTOHs和FOSAs检测和定量的响应和灵敏度更高。

1.2.2 气相色谱-质谱法

虽然LC-MS/MS也可用于检测FTOHs和FOSAs等化合物,但GC-MS/MS在半挥发性PFASs的分析应用更为广泛^[80,81,82,83]^。离子型PFASs因其自身不具有挥发性或者挥发性较弱,需衍生化处理后通过GC-MS测定。早在2009年,Chu和Letcher^[84]^结合固相萃取和衍生化处理后,经GC-MS测定了工业产品和环境生物样品中PFOS及其异构体;工业品中,除了直链全氟辛烷磺酸(L-PFOS)外,还鉴定出10种支链PFOS异构体;银鸥(*Larus argentatus*)卵、双冠鸬鹚(*Phalacrocorax auritus*)卵以及北极熊肝脏和血液中也检测到了L-PFOS和6种支链PFOS异构体;研究者利用该方法对猪肝样品中PFOS异构体的回收率、LOD等进行测定,发现除了L-PFOS的检出限为1.46 ng/mL,其余异构体LOD低至0.05~0.25 ng/mL。Gebbink等^[45]^以甲醇为萃取液,经衍生化处理后用GC-MS对2007年在北美劳伦山脉大湖地区15个群居地收集的银鸥蛋(*Larus argentatus*)中的PFOS异构体进行测定,结果显示PFOS同分异构体的LOD在0.01~0.94 ng/g ww,与Chu和Letcher^[84]^的结果相似;除此之外,L-PFOS在所有蛋中始终占据着异构体的主导地位,占∑PFOS浓度的95.0%至98.3%,这可能与直链PFOS较强的生物累积放大能力有关。

GC-MS检测挥发性PFASs的灵敏度较好,PFASs同类物尤其是同分异构体间的分辨率较高,并且基质效应的影响较低,但对其前处理要求却很高,如萃取浓缩过程中挥发性PFASs的损失、离子型PFASs离子衍生化的效率等,净化过程基质去除不完全等都会影响检测结果。因此目前LC-MS/MS仍是PFASs的主流检测方法。

## 2 鸟类蛋作为PFASs污染指示物的应用

鸟类蛋由蛋清和蛋黄组成,尽管研究显示,蛋黄中PFASs的检出率和浓度均要显著高于蛋清^[43]^,但由于蛋清和蛋黄之间的分离受到人为操作干扰较大,因此大部分研究都以蛋的整体作为研究基质。不同物种蛋间的组成成分相对一致^[85]^,且其中的污染物直接与污染物代际传输相关联^[86]^。禽蛋中的鸡蛋、鸭蛋等是人类重要的蛋白质来源。因此,蛋是环境科学研究中环境污染水平指示、污染物遗传发育毒性风险和摄入健康风险研究的理想介质。在对不同物种的蛋类进行对比后发现,鸡蛋中PFASs总体浓度远低于鸟蛋^[29,39,48,51,54]^,这可能与PFASs的生物放大效应相关。鸡和鸟的食物来源不同,鸡一般以喂食植物性饲料为主,而鸟一般在食物链占据较高的营养级。我们在PFASs点源区域的研究也发现,散养鸡蛋中PFASs浓度随着与氟化工厂的距离增加而降低^[87]^。这说明蛋类能够较好地指示物种和环境中PFASs的污染水平。

近年来,陆续有研究者通过使用蛋类作为非损伤性生物基质来评估生物体和生态系统的污染情况。PFASs在蛋类中的含量分布具有显著地区差异性。我们以PFASs中最受关注的PFOS为例,对涉及蛋类的现有研究进行了总结(见图2)。从整体来看,亚洲和环北极地区蛋中PFOS的浓度比北美洲和欧洲低1~2个数量级。来自美国密歇根州奥斯科达镇克拉克沼泽的树燕(*Tachycineta bicolor*)蛋中,PFOS平均含量为663 ng/g ww,占ΣPFSAs的90.1%,是美国鸟类PFASs污染浓度最高的;位于奥斯科达附近的空军基地消防演习中使用的泡沫灭火器可能是PFOS的潜在来源^[88]^。另外,在比利时安普卫特氟化工厂附近收集到的大山雀(*Parus Major*)蛋中也检测到了高浓度的PFOS,最高达48056 ng/g ww,但随着与工厂距离的增加,PFOS含量逐渐降低^[89]^。由此可见,PFASs的生产、使用及排放对周围的生态环境有着直接影响。

**图2:**
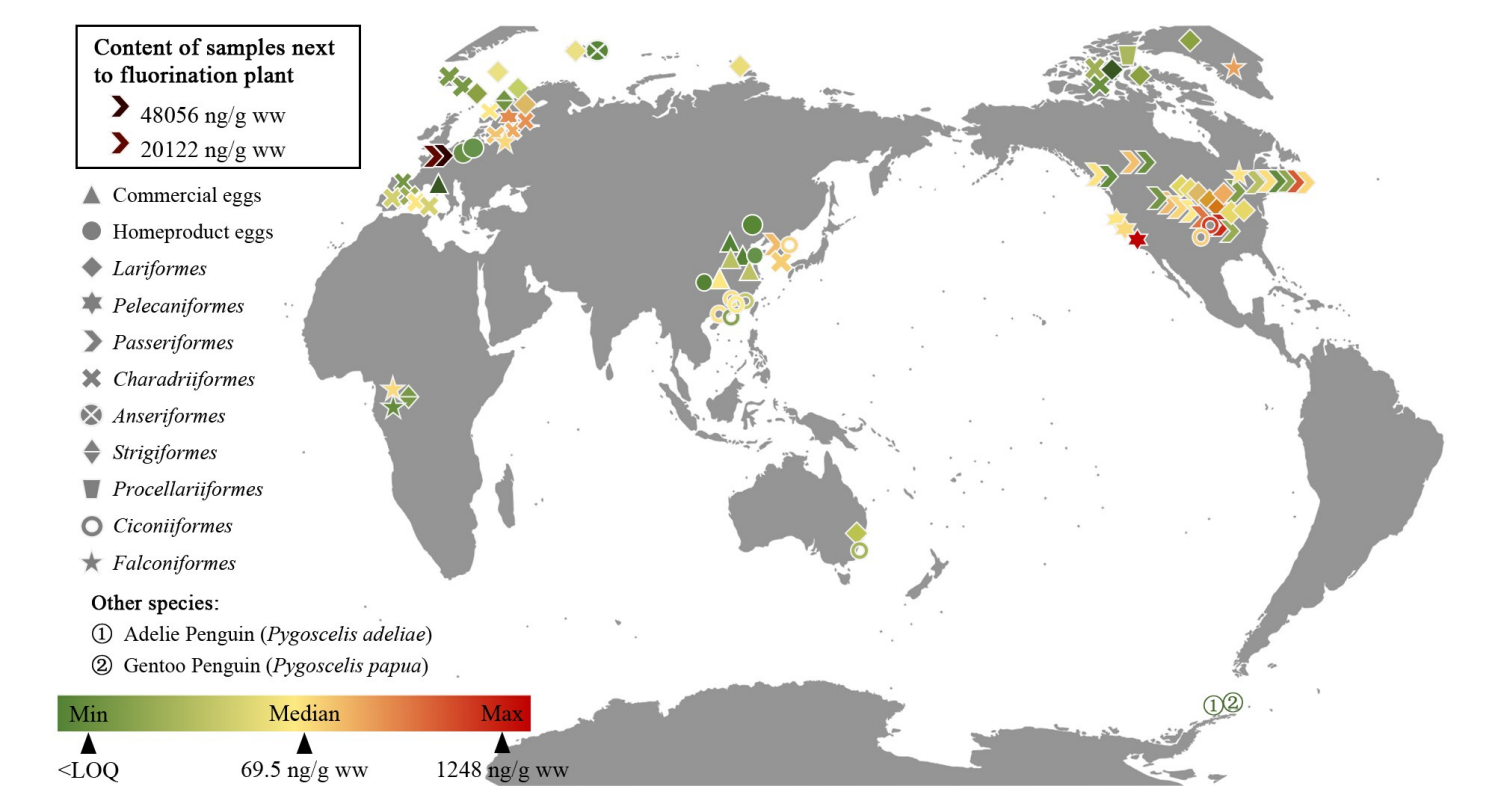
蛋中PFOS污染分布特征(基于国家测绘地理信息局标准地图服务网站下载的审图号为GS(2016)1561号的标准地图制作,底图无修改)

Yoo等^[27]^对韩国始华湖及其附近小白鹭(*Egretta garzetta*)、金眶鸻(*Charadrius dubius*)以及鹦鹉雀(*Paradoxornis webbiana*)这3类鸟蛋中的PFASs进行测定,将蛋类中PFOS的浓度与代表阈值的毒理学基准比较,评估了全湖中PFOS及其混合物对鸟类的生态风险,结果表明PFOS浓度尚不会对始华湖周围的鸟类产生不良影响。研究者们也开展了一系列以鸡蛋作为主要基质、将鸡蛋中PFASs污染水平与当地污染水平相联系并对人体健康造成的风险进行评估的工作。Su等^[29]^和Yuan等^[43]^分别研究了中国山东地区超市购买的鸡蛋中PFOS的含量,在前者的研究中鸡蛋内PFOS的浓度比后者低2个数量级。风险评价结果表明,目前中国鸡蛋中PFOS的污染水平不会对人类产生潜在健康危害^[34,40,43,46]^。但值得注意的是,不同地域鸡蛋中所检测的单体含量和特征不完全一致,这可能是由于喂养饲料习惯导致,因此对于通过摄入蛋类PFASs导致的人体健康风险评价仍有待更加系统和全面的研究。Braune等^[52]^利用加拿大北极地区海鸟蛋中的PFASs浓度对该区域不同物种PFASs污染水平和时间趋势进行了评估,厚嘴海鸥(*Uria lomvia*)和暴雪鹱(*Fulmarus glacialis*)在1975年到2011年之间∑PFCA的浓度显著增加,年增长分别为0.56 ng/g ww和0.91 ng/g ww,而PFOS的浓度没有明显变化。Eriksson等^[35]^也通过鸟蛋评估了PFASs的环境变化,瑞典游隼(*Falco peregrinus*)和鱼鹰(*Pandion haliaetus*)中的PFOS含量没有显著差异(1997至2001年103 ng/g、2008至2009年64 ng/g、2013年70 ng/g),尽管近20年来PFOS生产使用受到管制,但环境中没有出现预计中的大幅下降趋势。环境中PFOS持续的高水平可能与其较高的持久性以及历史产品中的缓慢释放有关^[2]^。

## 3 总结与展望

本文总结了PFASs在蛋类基质中的前处理、仪器分析方法,以及蛋作为PFASs污染指示物的发展现状。蛋类样品因其脂质含量丰富,生物样品基质效应明显,对一些基质的方法灵敏度仍显示出不足。因此在接下来进行蛋类PFASs的研究时,需要进一步优化样品前处理过程,有效分离并特异性富集待测物,从而减小基质影响,同时优化检测方法以适应痕量样品分析。

蛋类作为PFASs污染指示物的应用逐渐成熟,尤其是作为非损伤性生物基质,不仅便于偏远区域的采集、运输,而且对于研究濒临灭绝的野生动物提供了保护。已有研究表明,蛋类作为非损伤性生物基质可反映不同地区PFASs的污染程度,检测蛋类中PFASs污染水平可为区域环境PFASs污染评估提供更多的科学数据。同时蛋类也是人类日常膳食中不可或缺的动物源性食品,是日常营养摄入的重要组成部分,它可作为一种评估生态及人类健康的风险指示物。因此需加强蛋类中新型污染物的生物监测,进一步推广其作为环境污染指示物以应用于新型污染物的评估。
